# Rasch and Confirmatory Factor Analyses of the Arabic Version of the Diabetes Self-Management Scale (DSMS): An Intercultural Approach

**DOI:** 10.3390/healthcare11010035

**Published:** 2022-12-22

**Authors:** Yahia Ali Kaabi, Nahla A. Alshaikh, Ahmed A. Jerah, Mustafa A. Halawi, Mahmoud M. Habibullah, Siddig Ibrahim Abdelwahab

**Affiliations:** 1 Medical Research Center, Faculty of Medicine, Jazan University, Jazan 45041, Saudi Arabia; 2 Department of Medical Laboratory Technology, Faculty of Applied Medical Sciences, Jazan University, Jazan 42200, Saudi Arabia

**Keywords:** diabetes mellitus, compliance, diabetes self-management scale, Rasch model, psychometric testing, AMOS

## Abstract

The current study was designed to validate the Arabic version of the Diabetes Self-Management Scale (DSMS) using Rasch and confirmatory factor analyses. This included person and item fit, separation, and reliability; rating scale functionality to evidence substantive validity; unidimensional structure to evidence structural validity; and item technical quality to evidence content validity. The study was conducted between September 2021 and March 2022. Utilizing AMOS-based confirmatory factor analysis (CFA), the study also assured the dimensionality of the DSMS. The participants were 103 diabetic patients in Saudi Arabia with a mean age of 44.72 years (standard deviation = 17.35). The analysis was performed using a trichotomous rating scale, and only one item exhibited a misfit (DSMS14). The item difficulty range was −1.0 to +1.0 logits, while the person’s ability range was −3.0 to +3.0 logits. The first construct proved one Rasch dimension, which was explained and further analyzed using AMOS-CFA for the one-factor model. The DSMS was shown to be beneficial as a screening instrument for patient-reported diabetes self-management, despite several flaws that need to be addressed to improve the scale further.

## 1. Introduction

Diabetes is a chronic disease that affects a considerable portion of the global population. The morbidity rate is increasing year over year in addition to being widespread. In order to help persons with diabetes maintain glycemic control and avoid complications, diabetes self-care management is therefore seen as being vital in treating diabetes [1,2,3]. Patients diagnosed with diabetes are required to implement a rigorous and time-consuming self-management routine into their everyday life (e.g., taking medication, diet, exercise). Diabetes self-management (DSM) is essential to achieving diabetes control and warding off diabetic complications [4]. Diabetes mellitus has been demonstrated to have a tight connection to an increased risk of early and preventable death and a heightened risk of both macro- and microvascular consequences, such as stroke, blindness, end-stage renal failure, cardiovascular disease, and amputation. In addition, the treatment of diabetic people is highly expensive [5]. Proper glycemic control reduces the risk of diabetes complications and treatment costs. Someone’s glycemic control can be tested. A one percent drop in glycemic index was linked to a 37% reduction in diabetic complications and a 21% reduction in diabetes mortality. Prior research shows that type 2 diabetes self-management training is useful for short-term glucose regulation. Another study found that following DSM is essential to managing diabetes, and those who do so have better short- and long-term health. Diabetes self-management is key to overall management [5,6,7,8].

A contemporary test theory method for evaluating the psychometric qualities of instruments is the Rasch analysis. It may be applied while creating new instruments or assessing and improving ones that already exist [9]. The Rasch analysis looks at both the instrument’s components and its users. The main premise of a Rasch analysis is that a person’s ability and difficulty are connected to the likelihood of answering correctly or erroneously to a particular item [10]. The Rasch analysis provides numerous types of internal construct validity evidence, including unidimensionality, category function, and item and person separation [10].

There are several tools available to measure DSM. These tools may be made for use by patients or podiatrists and have various objectives and viewpoints. The majority of the instruments are diverse. Four instruments—the Summary of Diabetes Self-Care Activities (SDSCA), Confidence in Diabetes Self-Care (CIDS), Self-Care Inventory-Revised (SCI-R), and the Diabetes Self-Management Questionnaire (DSMQ)—are used to assess DSM, according to pertinent research. The SCI-R primarily focuses on assessing patients’ self-management behavior, whereas the SDSCA does not include items concerning teamwork and objectives in the illness management process. The DSMQ only applies to patients with a long duration and severity of illness, which is not reflective of the majority of patients with diabetes, while the CIDS lacks an evaluation of patients’ treatment objectives [11,12,13]. According to the instrument created by Gharaibeh et al., self-efficacy, self-care management, and self-care agency all have direct correlations [14].

For the development of research and the application of research results in practice, valid and reliable instruments are crucial [15]. In order to provide suitable treatments that can be successful in reaching the goals of diabetes management, it is also vital to establish accurate and reliable measures to assess the actual performance of diabetes self-management activities [16,17]. Thus, the Diabetes Self-Management Scale was created to assess diabetes self-care management (DSMS) [18,19]. However, the scale’s validity was not tested in Arabic-speaking communities. Therefore, the current study was designed to validate the Arabic version of the Diabetes Self-Management Scale (DSMS) using Rasch and confirmatory factor analyses to facilitate its adoption in research conducted in Arabic-speaking societies.

## 2. Materials and Methods

### 2.1. Study Design and Sample Size

A community-based cross-sectional design was adopted. The data were collected electronically. Random sampling was applied to collect the respondents’ feedback on the questionnaire. The recommended sample size for Rasch analysis is 100 respondents [20]. The study was conducted between September 2021 and March 2022. The data in the study were collected using a self-administered questionnaire. The data were collected electronically. The questionnaire was designed in Arabic to be suitable for the participants. The questionnaire yielded data about demographic characteristics such as gender, marital status, level of education, job, history of DM, type of treatment, age, weight, and height.

### 2.2. Instrument

The questionnaire on compliance with diabetes self-management, which was modified from the questionnaire developed by Park (1985), consists of a total of 14 questions [18,19,21]. Four of these questions concern diet control, two concern exercise, two concern insulin treatment, and the remaining five concern self-monitoring blood glucose and general management (Table 1). On a Likert scale ranging from 1 (always) to 4 (never), respondents were asked to assess their level of agreement with each statement, with higher scores suggesting less effective diabetes control. The research team translated the scale and checked it with a professional Arabic translator. The content validity was checked by two researchers.

### 2.3. Rasch Model Testing

Unidimensionality, item fit, person fit, person separation, and item hierarchy were assessed using the Rasch partial credit model. This study used WINSTEPS to analyze the data (Winsteps Rasch Measurement Analysis; Version 5.2.2.0, Chicago, IL, USA) [9,20].

### 2.4. Analysis

The rating scale’s functioning was examined to determine the number of response options by assessing category frequencies, average measures, infit and outfit mean squares, and threshold calibrations. There are a minimum of ten responses per category, and averages should increase monotonically [20]. If the rating scale has few category frequencies or disordered averages, some response categories may be combined [20].

Rasch requires unidimensionality. Unidimensional instruments measure a single construct across all items [22]. Unidimensionality can be assessed by looking at item fit statistics and performing a PCA of residuals [23]. Fit statistics identify items or participants whose responses were unexpected. Infit and outfit statistics report normalized mean square residuals. Outfit statistics are sensitive to unexpected responses far from an item’s measure [17]. Both infit and outfit statistics have two forms: MnSq and Zstd [22]. An MnSq value of 1.4 or a Zstd value of 2.0 indicates a misfit, meaning the item’s performance does not match the Rasch model’s expectations. Infit values below 0.6 and a Zstd of −2 suggest an item does not contribute independent information [22,23,24].

Principal component analysis was used to examine the internal validity of DSMS. The first component must explain more than 50% of the total variance for the instrument to be unidimensional [25]. Person-response validity is shown by person fit. This was evaluated by inspecting a person’s goodness-of-fit values. The criteria were 1.4 logits and 2 Zstd [26,27].

Person reliability and person separation measure how the tool distinguishes respondents. Items must be sufficiently different in difficulty to determine the latent scale’s direction and meaning [28,29]. The separation index measures how well the DSMS differentiates foot health levels. Separation index 2.0, reliability 0.80. Item reliability was calculated to determine whether item response categories reflected increasing levels of difficulty (item separation criterion of 2.0 and reliability of 0.80). Separation indices of 1.5, 2.0, and 3.0 divide respondents into two, three, and four strata, respectively [30]. Person separation equals reliability in classical test theory. In Rasch analysis, its meaning is not as pivotal, but the index shows the power of fit analysis [30].

The item hierarchy determines difficulty based on person ability. The Rasch model estimates item locations (calibrations) along a measurement continuum [31]. Item calibration ranks the severity of scale items. The Rasch model shows how well items fit a group [32]. Item calibration is described in log-odds units (logits), where a larger magnitude indicates item difficulty [31,33]. Ideal item distribution matches participant distribution [31,33,34,35,36,37,38,39,40,41]. This study evaluated item difficulty using an item map and a continuum. This is known as a Wright Map. It displays Rasch person and item measure data. Person and item measures use the same logit scale to plot them together. A Wright Map allows a researcher to examine the spread of items, the respondents’ location, and the test takers’ performance, utilizing the collection of test items to explain what skills a test taker has [34].

### 2.5. AMOS-Based Confirmatory Factor Analysis

The factor structure for the DSMS was tested using confirmatory factor analysis (CFA) in AMOS 24 (SPSS version 24). The AMOS-based model was assessed using the recommended threshold for model fit indices (Table 2). Modification indices were utilized for the correlation between unobserved variance (e1–e13). Assessment of model fit involves considering several indices of model fit, including root mean square error of approximation (RMSEA), comparative fit index (CFI), Chi-square/df (cmin/df), the *p*-value for the model, adjusted Good of fit (GFI), and standardized root mean square residual (SRMR).

### 2.6. Ethical Consideration

The entire course of the investigation was conducted according to the good scientific procedure. The local Jazan University Research Ethics Committee gave its permission to this study, which adhered to the values outlined in the Declaration of Helsinki. The project’s approval number was REC-43/02/017. The study’s goal, the fact that participation was optional, and the anonymity and confidentiality of the reporting were all explained in writing to each participant. Each participant signed a permission form after receiving full information.

## 3. Results

### 3.1. Description of the Participants

In total, responses from 103 diabetic patients were included in the Rasch analysis. The mean age of the participants was 44.72 years (range 18–95, SD 17.35). A total of 56.3% of the participants were male, while 43.7% of them were female. The majority were married (68.9%). Additionally, 40.8% of the sample possessed a university degree, while 51.5% of them were unemployed. History of DM diagnosis had almost similar responses (Table 1).

### 3.2. Function of the Rating Scale

To ensure that the DSMS rating scale was applied as intended, the performance of the rating scale was assessed. In this data set, 14 of the intended ten responses per category were not obtained for some of the options in the Likert scale of DSMS. The fourth option in the Likert scale was merged with the third one. This was done to obtain more reliable estimates of the item’s difficulty. The minimum and maximum responses were 12 and 61, respectively. In every question, every response option was used.

### 3.3. Item and Person Fit

Based on item fit values, all items except item 14 (I take my diabetic medication, such as insulin injection as prescribed, regularly observing dosage and time) had acceptable item fit statistics, and the loading fell within the permissible range (Table 3). The Zstd values ranged from −0.15 to 2.66, and the item MnSq ranged from 0.68 to 1.40. Most participants fit the Rasch model with a satisfactory level of goodness-of-fit. One person, though, stood out as odd.

### 3.4. Unidimensionality

The first component exhibited a good amount of unidimensionality, as it accounted for 33.1% of the variation in the data. Internal scale validity was supported by the fact that the greatest first contrast component explained 11.8% (eigenvalue 2.29), while the second largest explained 8.7% (eigenvalue 1.68) of the variation.

### 3.5. Separation Indexing and Reliability

**Figure 1:**
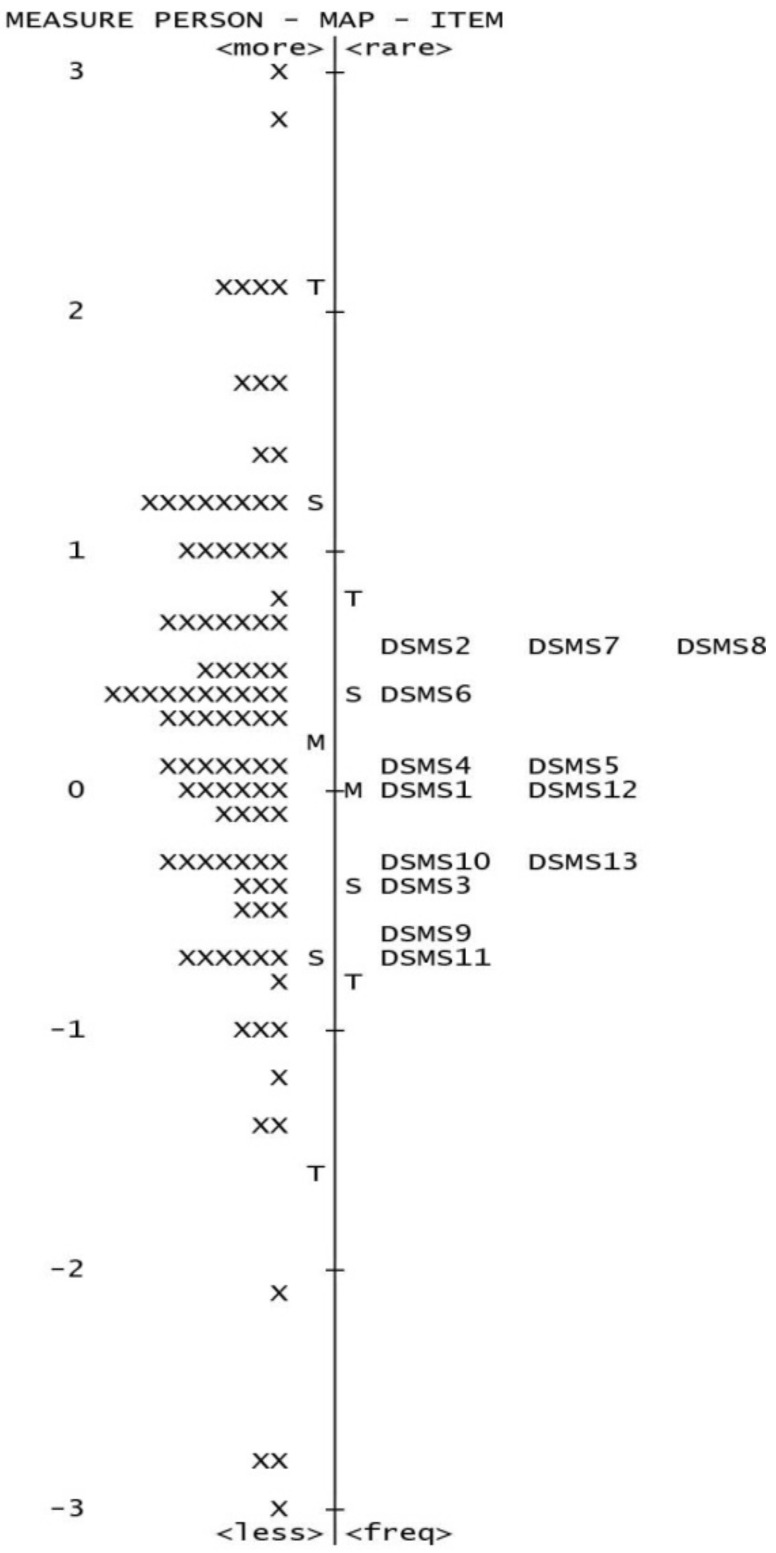
Item map.

The person separation index (1.68) indicated that the people who were included were sufficiently separated from one another. The device was able to distinguish between and separate the objects, as evidenced by the item separation figure of 2.73. The participant hierarchy (item map) shows how the participants and objects fit together on a continuum. The individuals in this research tended to be broader than the items. This suggests that the sample’s ability and the ability indicated in the items were related. The test-item targeting is good since the mean of the item measures was less than one standard deviation lower than the mean of the person measurements (Figure 1). The reliability for item and person is 0.88 and 0.76, respectively.

### 3.6. CFA

**Figure 2:**
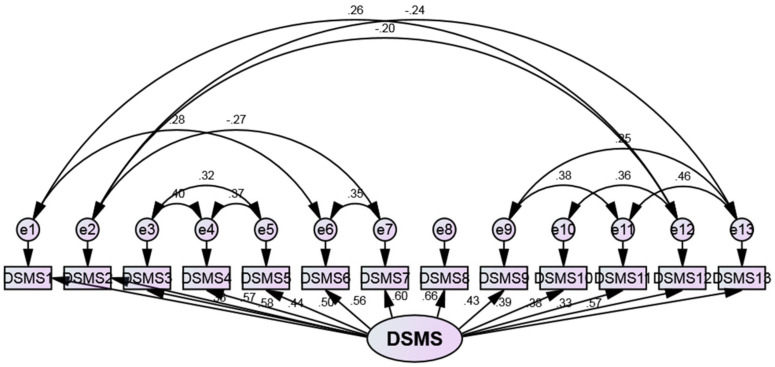
Confirmatory factor analysis. The analysis was conducted using AMOS software.

The results of the CFA can be seen in Table 2 and Figure 2, demonstrating that the overall goodness-of-fit Chi-square was used as a fit index for the model. Root mean square error of approximation (RMSEA), Comparative fit index (CFI), Chi-square/df (cmin/df), P-value for the model, Good of fit (GFI), and PCLOSE are all with the required threshold for the model fit. The created correlation between unobserved variance (e1–e13) based on the modification indices’ recommendations improved the data’s fitness to the model and achieved possible unidimensional DSMS. 

## 4. Discussion

This study investigated the DSMS psychometrics using item response theory and a Rasch analysis on diabetes patients’ replies. This cohort’s DSMS results were mixed. All items except item 14 had adequate internal scale validity in terms of unidimensionality. Removing one participant cured the person’s fit. Rasch analysis has been applied previously in various cultural media, including the Arabic versions of various scales [35,36,37]. The current findings are encouraging, particularly when we consider the difficulty of defining the concept of DSMS and developing effective measurement tools to capture it.

Rasch analysis was conducted using a modified rating scale, with the original categories collapsed into a trichotomous scale because some items lacked sufficient responses in all response categories [20,38]. This small sample size did not appear to be an issue, as the item separation index confirmed that the number of participants in our study was sufficient for testing the scale items [25]. Some categories may have a low response rate for reasons other than the sample size, such as when participants need help distinguishing between multiple categories. Additional research is required to confirm whether respondents underutilize some categories on the original rating scale.

Item 14, “I take my diabetes medication like insulin injection as prescribed, observing dosage and time, regularly,” was the most unfitted statement in the DSMS scale as observed by its unsatisfactory MnSq and Zstd. It is concerning as prior research has confirmed that ambulatory persons with diabetes frequently self-administer insulin incorrectly. These studies strongly advise that patients’ correct insulin self-administration practices should be reviewed and retaught more regularly. Patients with lower incomes and educational levels may benefit the most from this [39,40]. Based on this, future research should concentrate on enhancing the procedures and the frequency of patient education to prevent errors, hopefully reducing both episodes of hyperglycemia and hypoglycemia [39]. In future studies, the current way of measuring how well people take their medicine should be looked at in more depth.

The first component exhibited a good amount of unidimensionality, as it accounted for 33.1% of the variation in the data. The fact that the percentage does not exceed 50% may be due to the fact that the items on the scale are related to food and meal management, medication adherence, regular blood sugar testing, and doctor’s appointments. Developing a multidimensional tool with separate items for each dimension is more efficient. This increases the percentage of varying interpretations. In the current study, however, Rasch’s analysis revealed that the scale was unidimensional (eigenvalue = 2.29) based on the reported guidelines [41]. The results of the CFA demonstrate that the overall goodness-of-fit Chi-square was used as a fit index for the model (Table 2). The created correlation between unobserved variance (e1–e13) based on the modification indices’ recommendations improved the data’s fitness to the model and achieved possible unidimensional DSMS. The confirmatory factor analysis results were used previously to support the Rasch-obtained findings [32]. 

## 5. Conclusions

Researchers in the Arab region who are interested in diabetes self-management compliance will find this study useful because it gives an assessment instrument. This is of utmost significance given the scarcity of validated measures that have been examined using a variety of samples. The fact that very few scales have been established in Arabic and evaluated on a variety of samples bolsters the significance of this study even more. This is particularly true for scales that concentrate on topics that are relevant to people who are afflicted with diabetic conditions. A deeper knowledge of the psychometric features of this scale can be attained by examining it with diverse samples from the Arab area and other parts of Saudi Arabia.

## Figures and Tables

**Table 1:** Socio-demographic characteristics of respondents.

Variables	Categories	N	%
Gender	Male	58	56.3
	Female	45	43.7
Marital status	Married	71	68.9
	Single	32	31.1
Level of Education	Primary School	16	15.5
	Intermediate	13	12.6
	High School	32	31.1
	University	42	40.8
Job	Student	8	7.8
	Employed	42	40.8
	Unemployed	53	51.5
History of DM	Less than one year	17	16.5
	Less than two years	16	15.5
	Less than three years	11	10.7
	Less than five years	20	19.4
	Less than ten years	22	21.4
	Less than twenty years	13	12.6
	Less than thirty years	4	3.9
Type of treatment	Diet	23	22.3
	Diet + Metformin	41	39.8
	Diet + Metformin + insulin	39	37.9
Age	44.72 ± 17.35		
Weight	77.01 ± 19.86		
Height	161.84 ± 13.96		

**Table 2:** Assessment of model fit using AMOS and CFA.

Measure	Threshold	DSMS Performance in CFA
Root mean square error of approximation (RMSEA)	>0.05	0.046
Comparative fit index (CFI)	>0.95	0.969
Chi-sqaure/df (cmin/df)	<3.0	1.213
P-value for the model	>0.05	0.140
Good of fit (GFI)	>0.95	0.926
PCLOSE	>0.05	0.545

**Table 3:** Item fit statistics.

Abbreviated Items	Abbreviation	Measure	SE	Infit		Outfit	
				MnSq	Zstd	MnSq	Zstd
Adherence to doctor’s appointments	DSMS1	0.40	0.15	1.33	2.66	1.31	2.58
Taking foods regularly	DSMS2	0.65	0.15	0.94	−0.51	0.9	−0.66
Well-balanced diet	DSMS3	−0.40	0.15	0.78	−1.86	0.77	−1.42
Healthy food	DSMS4	0.17	0.14	1.06	0.55	1.15	1.04
Controlling salt	DSMS5	0.08	0.14	0.97	−0.21	1.01	0.14
Blood sugar test	DSMS6	0.44	0.15	0.85	−1.36	0.89	−0.76
Blood sugar test when needed	DSMS7	0.52	0.15	0.98	−0.15	0.96	−0.26
Maintaining the optimal blood-sugar level	DSMS8	0.63	0.15	0.68	−2.09	0.68	−2.41
Controlling the size of meals or exercise	DSMS9	−0.62	0.16	0.88	−0.89	0.89	−0.51
Emergency foods	DSMS10	−0.4	0.15	1.12	0.96	1.12	0.74
Maintaining optimal weight	DSMS11	−0.69	0.16	1.00	0.06	1.08	0.44
Readiness for trips	DSMS12	−0.06	0.15	1.30	2.37	1.31	1.95
Attending educational programs	DSMS13	−0.33	0.15	0.91	−0.73	0.90	−0.59

## Data Availability

Data are available upon request.
